
JOURNAL INFORMATION
==============================
NLM Title Abbreviation: ScientificWorldJournal
Journal ID: ScientificWorldJournal
NLM Journal ID: 101131163
Journal ID: TSWJ

The Scientific World Journal

ISSN: 2356-6140
EISSN: 1537-744X
Publisher: Wiley

ARTICLE INFORMATION
==============================
PMCID: PMC5823087
PMID: 19347236
DOI: 10.1100/tsw.2009.33
Article ID: 567564
Article version: 1
Subjects: Directions in Science

Impact of Mitochondrial Dynamics on Organismic Aging

Scheckhuber Christian Q.
Johann Wolfgang Goethe University, Cluster of Excellence Macromolecular Complexes and Faculty for Biosciences, Molecular Developmental Biology, Max-von-Laue-Str. 9, 60438 Frankfurt/Main, Germany
*Christian Q. Scheckhuber: scheckhuber@bio.uni-frankfurt.deAcademic Editor: Rudy Tanzi

Publication date: 2009
Electronic publication date: 2009 Apr 1
Volume: 9
First page: 250
Last page: 254
Received 2009 Feb 3; Revised 2009 Mar 27; Accepted 2009 Mar 29
Copyright: Copyright © 2009 Christian Q. Scheckhuber.
Copyright year: 2009
License: This is an open access article distributed under the Creative Commons Attribution License, which permits unrestricted use, distribution, and reproduction in any medium, provided the original work is properly cited.
License URL: https://creativecommons.org/licenses/by/3.0/

Keywords: mitochondria, mitochondrial dynamics, fusion, fission, Podospora anserina, aging, autophagy, reactive oxygen species

Funding: European Commission LSHM-CT-2004-512020

==============================
Directions in Science
*TheScientificWorldJOURNAL* (2009) 9, 250–254
ISSN 1537-744X; DOI 10.1100/tsw.2009.33

### Impact of Mitochondrial Dynamics on Organismic Aging

Christian Q. Scheckhuber

*Johann Wolfgang Goethe University, Cluster of Excellence Macromolecular Complexes and Faculty for Biosciences, Molecular Developmental Biology, Max-von-Laue-Str. 9, 60438 Frankfurt/Main, Germany*

**E-mail**: scheckhuber@bio.uni-frankfurt.de

*Received February 3, 2009; Revised March 27, 2009; Accepted March 29, 2009; Published April 1, 2009*

---

**KEYWORDS**: mitochondria, mitochondrial dynamics, fusion, fission, *Podospora anserina*, aging, autophagy, reactive oxygen species

---

Biological aging is accompanied by progressive and irreversible impairments of physiological functionality with concomitant increases in morbidity and mortality. Aging is governed by an intricate network of various molecular pathways that affect the cell. In many eukaryotic organisms studied so far, the powerhouses of the cell, mitochondria, are, on the one hand, mandatory to keep the cell alive due to a number of metabolic activities. On the other hand, these organelles were found to play a crucial role in processes like apoptosis and aging. While the former is initiated by the release of death-inducing factors like cytochrome-c, "apoptosis-inducing factor" (AIF), endonuclease G, and Smac/DIABLO from the mitochondria[1], reactive oxygen species (ROS) formed during respiratory activity are among the key factors that contribute to cellular aging, according to the "Mitochondrial Theory of Aging"[2]. Therefore, mitochondria have been the target of dedicated research in order to understand the cellular pathways these organelles effect and how their functionality is regulated[3,4].

An important regulatory factor of mitochondrial function is the dynamic morphology transitions of these organelles. Spherical mitochondria can be conveniently distributed within a cell and segregated to daughter cells. Mitochondrial fusion is essential during embryonic development and biogenesis of sperm cells in *Drosophila melanogaster*[5], and is important for content mixing of mitochondria[6]. Compounds such as metabolites, oxygen, proteins, lipids, and mtDNA can be efficiently transferred in the mitochondrial compartment. Mitochondrial fusion was shown to enable the complementation of mtDNA defects[7,8]. Moreover, the inner membrane potential ($\Delta\Psi_M$) can also be transmitted between fusing mitochondria, allowing "electric coupling" and efficient transfer of energy[9]. Groundbreaking insights into how mitochondrial morphological transitions are regulated have been gained from research on baker's yeast *Saccharomyces cerevisiae*[10,11]. In yeast, fission is mainly performed by Dnm1p, Mdv1p, and Fis1p[12,13,14,15,16]. Fis1p is located in the outer mitochondrial membrane and interacts with Mdv1p. Mdv1p binds to Dnm1p via a WD40 domain. Dnm1p is a large GTPase, which is regarded as the "master regulator of mitochondrial fission" in yeast[10]. Homologs of Dnm1p have been identified in various organisms, including nematodes[17], flies[18], and mammals[19], suggesting an evolutionarily conserved mechanism of mitochondrial division. The molecular machinery executing mitochondrial fusion in yeast consists of Fzo1p, Ugo1p, and Mgm1p, in addition to regulatory proteins. Fzo1p is a large GTPase situated in the outer mitochondrial membrane, which is needed for tethering and outer-membrane

©2009 with author.

fusion of juxtaposed mitochondria[20,21,22,23,24]. The exact role of the outer membrane protein Ugo1p in the fusion process has not been clearly elucidated so far. Mgm1p belongs to the class of large GTPases and has been shown to be needed for inner membrane fusion and remodeling of the cristae membranes[22,25]. The human orthologue of Mgm1, OPA-1, is known to have different splice variants that can be proteolytically processed at different sites to generate different isoforms[26,27,28,29,30,31]. Importantly, OPA-1 deficiency and mutation, respectively, are associated with a number of diseases (e.g., ADOA [autosomal dominant optic atrophy], ataxia, deafness, multiple sclerosis–like disorders)[32,33,34,35].

Mitochondrial morphology transitions have been recently shown to affect the aging of fungal model systems such as yeast and the filamentous ascomycete *Podospora anserina,* which has been a convenient model organism for the study of aging for more than 50 years[36,37,38].

The *P. anserina* mutant *PaDnm1*::ble, which is impaired in mitochondrial fission due to the deletion of the fission gene *PaDnm1*, has been characterized in recent studies[39,40]. Interestingly, in contrast to most other *P. anserina* longevity mutants, *PaDnm1*::ble does not display phenotypic defects (e.g., slow growth rate, reduced fertility, sterility). Therefore, the healthy period of the lifetime, the health span, is extended in *PaDnm1*::ble. In *PaDnm1*::ble, the normally short filamentous mitochondria appear to be highly elongated and in some cases interconnected[39]. On standard complex growth medium, *PaDnm1*::ble isolates benefit from a highly increased mean lifespan (244 vs. 22 days wild-type). Factors proposed to be responsible for the beneficial effect on aging are (1) a stabilized mitochondrial genome, (2) delayed fragmentation of mitochondria, (3) decreased ROS generation, and (4) increased resistance to apoptosis stimulation[39,40]. The last point suggests that the activation of recently identified apoptotic components like metacaspases in the terminal stage of the *P. anserina* life[41,42] is also delayed in this particular mutant. Significantly, elevated resistance against apoptotic stimulation has also been demonstrated in Dnm1/Drp1 mutants of yeast[43], *Caernorhabditis elegans*[44], *D. melanogaster*[45,46], and mammalian cell lines[47,48,49,50] in which the gene that encodes the corresponding PaDNM1 orthologue has been deleted or down-regulated. Collectively, the studies show that *PaDnm1*::ble not only displays an extended life span, but also a prolonged health span, underlining that *P. anserina* is a suitable model organism to study molecular pathways leading to healthy aging.

In addition to apoptosis regulation, the control of mitochondrial dynamics has recently been identified to be important for processes that might also play vital roles for aging, autophagy of dysfunctional mitochondria (mitophagy), and resistance to ROS, respectively. Mitophagy is regarded as a pathway to recycle dysfunctional or damaged mitochondria[51]. Therefore, mitophagy plays an important role for the quality control of mitochondria. Recently, mitochondria were found to divide asymmetrically in a Drp1-dependent manner[52]. One mitochondrion retained its normal membrane potential and was able to fuse with other mitochondria, whereas the other had a lowered $\Delta\Psi_M$ and decreased levels of the fusion protein OPA-1[52]. This way, the damaged mitochondrion was removed from the mitochondrial population, increasing the chance for its degradation by the autophagosome. At present, it is unclear whether this intriguing mechanism is decreased during aging and if this could also account for increased levels of dysfunctional mitochondria in old cells.

The *C. elegans* homologue of OPA-1, EAT-3, was identified as an essential factor for resistance against ROS[53]. If in loss-of-function mutants of *eat3,* the *sod2* gene, encoding a mitochondrial superoxide dismutase, is down-regulated, phenotypic defects like decreased brood size are enhanced. Moreover, the *eat3* mutants are also much more sensitive to the addition of a metabolic generator of superoxide anions[53]. In a mammalian cell line, it was shown that transient treatment with the ROS $H_2O_2$ impairs mitochondrial dynamics and that in response to this stress, an up-regulation of various fusion and fission genes at the transcript level was found[54]. These intriguing results connect the regulation of mitochondrial morphology to the defense against oxidative stress, which might constitute a new link in the complex network that regulates aging at the cellular level.

The identification and characterization of novel cellular pathways that might bear the potential to increase the healthy period of life is one of the desired aims of experimental aging research. Mitochondrial dynamics regulation has emerged as a candidate for achieving this goal, at least in two fungal model systems, *S. cerevisiae* and *P. anserina*. It will certainly be interesting to see whether or not these molecular pathways also play similar roles in higher biological systems.

### ACKNOWLEDGMENTS

The author wishes to thank Prof. Dr. Heinz D. Osiewacz (Johann W. Goethe University, Frankfurt, Germany) for his support. The experimental research of the author was funded by the European Commission via the Integrated Project with the acronym MiMage (LSHM-CT-2004-512020).

### REFERENCES

1. Taylor, R.C., Cullen, S.P., and Martin, S.J. (2008) Apoptosis: controlled demolition at the cellular level. *Nat. Rev. Mol. Cell Biol.* **9,** 231–241.
2. Harman, D. (1972) The biologic clock: the mitochondria? *J. Am. Geriatr. Soc.* **20,** 145–147.
3. Wallace, D.C. (2001) A mitochondrial paradigm for degenerative diseases and ageing. *Novartis Found. Symp.* **235,** 247–263.
4. Osiewacz, H.D. (2002) Mitochondrial functions and aging. *Gene* **286,** 65–71.
5. Hales, K.G. and Fuller, M.T. (1997) Developmentally regulated mitochondrial fusion mediated by a conserved, novel, predicted GTPase. *Cell* **90,** 121–129.
6. Busch, K.B., Bereiter-Hahn, J., Wittig, I., Schägger, H., and Jendrach, M. (2006) Mitochondrial dynamics generate equal distribution but patchwork localization of respiratory Complex I. *Mol. Membr. Biol.* **23,** 509–520.
7. Nakada, K., Inoue, K., Ono, T., Isobe, K., Ogura, A., Goto, Y.I., Nonaka, I., and Hayashi, J.I. (2001) Inter-mitochondrial complementation: mitochondria-specific system preventing mice from expression of disease phenotypes by mutant mtDNA. *Nat. Med.* **7,** 934–940.
8. Ono, T., Isobe, K., Nakada, K., and Hayashi, J.I. (2001) Human cells are protected from mitochondrial dysfunction by complementation of DNA products in fused mitochondria. *Nat. Genet.* **28,** 272–275.
9. Skulachev, V.P. (2001) Mitochondrial filaments and clusters as intracellular power-transmitting cables. *Trends Biochem. Sci.* **26,** 23–29.
10. Hoppins, S., Lackner, L., and Nunnari, J. (2007) The machines that divide and fuse mitochondria. *Annu Rev Biochem* **76,** 751–780.
11. Westermann, B. (2008) Molecular machinery of mitochondrial fusion and fission. *J. Biol. Chem.* **283,** 13501–13505.
12. Yoon, Y., Krueger, E.W., Oswald, B.J., and McNiven, M.A. (2003) The mitochondrial protein hFis1 regulates mitochondrial fission in mammalian cells through an interaction with the Dynamin-Like Protein DLP1. *Mol. Cell. Biol.* **23,** 5409.
13. James, D.I., Parone, P.A., Mattenberger, Y., and Martinou, J.C. (2003) hFis1, a novel component of the mammalian mitochondrial fission machinery. *J. Biol. Chem.* **278,** 36373–36379.
14. Mozdy, A.D., McCaffery, J.M., and Shaw, J.M. (2000) Dnm1p GTPase-mediated mitochondrial fission is a multi-step process requiring the novel integral membrane component Fis1p. *J. Cell Biol.* **151,** 367–380.
15. Tieu, Q. and Nunnari, J. (2000) Mdv1p is a WD repeat protein that interacts with the dynamin-related GTPase, Dnm1p, to trigger mitochondrial division. *J. Cell Biol.* **151,** 353–366.
16. Tieu, Q., Okreglak, V., Naylor, K., and Nunnari, J. (2002) The WD repeat protein, Mdv1p, functions as a molecular adaptor by interacting with Dnm1p and Fis1p during mitochondrial fission. *J. Cell Biol.* **158,** 445–452.
17. Labrousse, A.M., Zappaterra, M.D., Rube, D.A., and van der Bliek, A.M. (1999) *C. elegans* dynamin-related protein DRP-1 controls severing of the mitochondrial outer membrane. *Mol. Cell* **4,** 815–826.
18. Verstreken, P., Ly, C.V., Venken, K.J., Koh, T.W., Zhou, Y., and Bellen, H.J. (2005) Synaptic mitochondria are critical for mobilization of reserve pool vesicles at Drosophila neuromuscular junctions. *Neuron* **47,** 365–378.
19. Smirnova, E., Griparic, L., Shurland, D.L., and van der Bliek, A.M. (2001) Dynamin-related protein Drp1 is required for mitochondrial division in mammalian cells. *Mol. Biol. Cell* **12,** 2245–2256.
20. Wong, E.D., Wagner, J.A., Gorsich, S.W., McCaffery, J.M., Shaw, J.M., and Nunnari, J. (2000) The dynamin-related GTPase, Mgm1p, is an intermembrane space protein required for maintenance of fusion competent mitochondria. *J. Cell Biol.* **151,** 341–352.
21. Sesaki, H. and Jensen, R.E. (2004) Ugo1p links the Fzo1p and Mgm1p GTPases for mitochondrial fusion. *J. Biol. Chem.* **279,** 28298–28303.
22. Sesaki, H., Southard, S.M., Yaffe, M.P., and Jensen, R.E. (2003) Mgm1p, a dynamin-related GTPase, is essential for fusion of the mitochondrial outer membrane. *Mol. Biol. Cell* **14,** 2342–2356.
23. Coonrod, E.M., Karren, M.A., and Shaw, J.M. (2007) Ugo1p is a multipass transmembrane protein with a single carrier domain required for mitochondrial fusion. *Traffic* **8,** 500–511.

24. Wong, E.D., Wagner, J.A., Scott, S.V., Okreglak, V., Holewinske, T.J., Cassidy-Stone, A., and Nunnari, J. (2003) The intramitochondrial dynamin-related GTPase, Mgm1p, is a component of a protein complex that mediates mitochondrial fusion. *J. Cell Biol.* **160,** 303–311.
25. Meeusen, S., DeVay, R., Block, J., Cassidy-Stone, A., Wayson, S., McCaffery, J.M., and Nunnari, J. (2006) Mitochondrial inner-membrane fusion and crista maintenance requires the dynamin-related GTPase Mgm1. *Cell* **127,** 383–395.
26. Ishihara, N., Fujita, Y., Oka, T., and Mihara, K. (2006) Regulation of mitochondrial morphology through proteolytic cleavage of OPA1. *EMBO J.* **25,** 2966–2977.
27. Duvezin-Caubet, S., Jagasia, R., Wagener, J., Hofmann, S., Trifunovic, A., Hansson, A., Chomyn, A., Bauer, M.F., Attardi, G., Larsson, N.G., Neupert, W., and Reichert, A.S. (2006) Proteolytic processing of OPA1 links mitochondrial dysfunction to alterations in mitochondrial morphology. *J. Biol. Chem.* **281,** 37972–37979.
28. Duvezin-Caubet, S., Koppen, M., Wagener, J., Zick, M., Israel, L., Bernacchia, A., Jagasia, R., Rugarli, E.I., Imhof, A., Neupert, W., Langer, T., and Reichert, A.S. (2007) OPA1 processing reconstituted in yeast depends on the subunit composition of the m-AAA protease in mitochondria. *Mol. Biol. Cell* **18,** 3582–3590.
29. Olichon, A., Baricault, L., Gas, N., Guillou, E., Valette, A., Belenguer, P., and Lenaers, G. (2003) Loss of OPA1 perturbates the mitochondrial inner membrane structure and integrity, leading to cytochrome c release and apoptosis. *J. Biol. Chem.* **278,** 7743–7746.
30. Olichon, A., Elachouri, G., Baricault, L., Delettre, C., Belenguer, P., and Lenaers, G. (2007) OPA1 alternate splicing uncouples an evolutionary conserved function in mitochondrial fusion from a vertebrate restricted function in apoptosis. *Cell Death Differ.* **14,** 682–692.
31. Delettre, C., Griffoin, J.M., Kaplan, J., Dollfus, H., Lorenz, B., Faivre, L., Lenaers, G., Belenguer, P., and Hamel, C.P. (2001) Mutation spectrum and splicing variants in the OPA1 gene. *Hum. Genet.* **109,** 584–591.
32. Alexander, C., Votruba, M., Pesch, U.E., Thiselton, D.L., Mayer, S., Moore, A., Rodriguez, M., Kellner, U., Leo-Kottler, B., Auburger, G., Bhattacharya, S.S., and Wissinger, B. (2000) OPA1, encoding a dynamin-related GTPase, is mutated in autosomal dominant optic atrophy linked to chromosome 3q28. *Nat. Genet.* **26,** 211–215.
33. Verny, C., Loiseau, D., Scherer, C., Lejeune, P., Chevrollier, A., Gueguen, N., Guillet, V., Dubas, F., Reynier, P., Amati-Bonneau, P., and Bonneau, D. (2008) Multiple sclerosis-like disorder in OPA1-related autosomal dominant optic atrophy. *Neurology* **70,** 1152–1153.
34. Liguori, M., La Russa, A., Manna, I., Andreoli, V., Caracciolo, M., Spadafora, P., Cittadella, R., and Quattrone, A. (2008) A phenotypic variation of dominant optic atrophy and deafness (ADOAD) due to a novel OPA1 mutation. *J. Neurol.* **255,** 127–129.
35. Hudson, G., Amati-Bonneau, P., Blakely, E.L., Stewart, J.D., He, L., Schaefer, A.M., Griffiths, P.G., Ahlqvist, K., Suomalainen, A., Reynier, P., McFarland, R., Turnbull, D.M., Chinnery, P.F., and Taylor, R.W. (2008) Mutation of OPA1 causes dominant optic atrophy with external ophthalmoplegia, ataxia, deafness and multiple mitochondrial DNA deletions: a novel disorder of mtDNA maintenance. *Brain* **131,** 329–337.
36. Osiewacz, H.D. and Scheckhuber, C.Q. (2006) Impact of ROS on ageing of two fungal model systems: *Saccharomyces cerevisiae* and *Podospora anserina*. *Free Radic. Res.* **40,** 1350–1358.
37. Scheckhuber, C.Q. and Osiewacz, H.D. (2008) *Podospora anserina*: a model organism to study mechanisms of healthy ageing. *Mol. Genet. Genomics* **280,** 365–374.
38. Osiewacz, H.D. (1990) Molecular analysis of aging processes in fungi. *Mutat. Res.* **237,** 1–8.
39. Scheckhuber, C.Q., Erjavec, N., Tinazli, A., Hamann, A., Nyström, T., and Osiewacz, H.D. (2007) Reducing mitochondrial fission results in increased life span and fitness of two fungal ageing models. *Nat. Cell Biol.* **9,** 99–105.
40. Scheckhuber, C.Q., Rödel, E., and Wüstehube, J. (2008) Regulation of mitochondrial dynamics - characterization of fusion and fission genes in the ascomycete *Podospora anserina*. *Biotechnol. J.* **3,** 781–790.
41. Hamann, A., Brust, D., and Osiewacz, H.D. (2007) Deletion of putative apoptosis factors leads to lifespan extension in the fungal ageing model *Podospora anserina*. *Mol. Microbiol.* **65,** 948–958.
42. Hamann, A., Brust, D., and Osiewacz, H.D. (2008) Apoptosis pathways in fungal growth, development and ageing. *Trends Microbiol.* **16,** 276–283.
43. Fannjiang, Y., Cheng, W.C., Lee, S.J., Qi, B., Pevsner, J., McCaffery, J.M., Hill, R.B., Basanez, G., and Hardwick, J.M. (2004) Mitochondrial fission proteins regulate programmed cell death in yeast. *Genes Dev.* **18,** 2785–2797.
44. Jagasia, R., Grote, P., Westermann, B., and Conradt, B. (2005) DRP-1-mediated mitochondrial fragmentation during EGL-1-induced cell death in *C. elegans*. *Nature* **433,** 754–760.
45. Abdelwahid, E., Yokokura, T., Krieser, R.J., Balasundaram, S., Fowle, W.H., and White, K. (2007) Mitochondrial disruption in Drosophila apoptosis. *Dev. Cell* **12,** 793–806.
46. Goyal, G., Fell, B., Sarin, A., Youle, R.J., and Sriram, V. (2007) Role of mitochondrial remodeling in programmed cell death in *Drosophila melanogaster*. *Dev. Cell* **12,** 807–816.
47. Estaquier, J. and Arnoult, D. (2007) Inhibiting Drp1-mediated mitochondrial fission selectively prevents the release of cytochrome c during apoptosis. *Cell Death Differ.* **14,** 1086–1094.
48. Frank, S., Gaume, B., Bergmann-Leitner, E.S., Leitner, W.W., Robert, E.G., Catez, F., Smith, C.L., and Youle, R.J. (2001) The role of dynamin-related protein 1, a mediator of mitochondrial fission, in apoptosis. *Dev. Cell* **1,** 515–525.
49. Lee, Y.J., Jeong, S.Y., Karbowski, M., Smith, C.L., and Youle, R.J. (2004) Roles of the mammalian mitochondrial fission and fusion mediators Fis1, Drp1, and Opa1 in apoptosis. *Mol. Biol. Cell* **15,** 5001–5011.

50. Sugioka, R., Shimizu, S., and Tsujimoto, Y. (2004) Fzo1, a protein involved in mitochondrial fusion, inhibits apoptosis. *J. Biol. Chem.* **279,** 52726–52734.
51. Tatsuta, T. and Langer, T. (2008) Quality control of mitochondria: protection against neurodegeneration and ageing. *EMBO J.* **27,** 306–314.
52. Twig, G., Elorza, A., Molina, A.J., Mohamed, H., Wikstrom, J.D., Walzer, G., Stiles, L., Haigh, S.E., Katz, S., Las, G., Alroy, J., Wu, M., Py, B.F., Yuan, J., Deeney, J.T., Corkey, B.E., and Shirihai, O.S. (2008) Fission and selective fusion govern mitochondrial segregation and elimination by autophagy. *EMBO J.* **27,** 433–446.
53. Kanazawa, T., Zappaterra, M.D., Hasegawa, A., Wright, A.P., Newman-Smith, E.D., Buttle, K.F., McDonald, K., Mannella, C.A., and van der Bliek, A.M. (2008) The *C. elegans* Opa1 homologue EAT-3 is essential for resistance to free radicals. *PLoS Genet.* **4,** e1000022.
54. Jendrach, M., Mai, S., Pohl, S., Vöth, M., and Bereiter-Hahn, J. (2008) Short- and long-term alterations of mitochondrial morphology, dynamics and mtDNA after transient oxidative stress. *Mitochondrion* **8,** 293–304.

**This article should be cited as follows:**

Scheckhuber, C.Q. (2009) Impact of mitochondrial dynamics on organismic aging. *TheScientificWorldJOURNAL* **9**, 250–254. DOI 10.1100/tsw.2009.33.
